# Receptivity of providing firearm safety storage devices to parents along with firearms safety education

**DOI:** 10.3389/fpubh.2024.1352400

**Published:** 2024-03-21

**Authors:** Kiesha Fraser Doh, Zhana Bishop, Trishanne Gillings, Jonathan Johnson, Angela Boy, Rabbia S. Waris, Amina M. Bhatia, Matthew T. Santore, Harold K. Simon

**Affiliations:** ^1^Children's Healthcare of Atlanta, Atlanta, GA, United States; ^2^Department of Pediatrics, Emory University School of Medicine, Atlanta, GA, United States; ^3^Department of Emergency Medicine, Emory University School of Medicine, Atlanta, GA, United States; ^4^Emory University School of Medicine, Atlanta, GA, United States; ^5^Department of Surgery, Emory University School of Medicine, Atlanta, GA, United States

**Keywords:** safe storage, firearm, pediatric, gun, safety device

## Abstract

**Background:**

In the United States, 33% of households with children contain firearms, however only one-third reportedly store firearms securely. It’s estimated that 31% of unintentional firearm injury deaths can be prevented with safety devices. Our objective was to distribute safe storage devices, provide safe storage education, evaluate receptivity, and assess impact of intervention at follow-up.

**Method:**

At five independent, community safety events, parents received a safe storage device after completing a survey that assessed firearms storage methods and parental comfort with discussions regarding firearm safety. Follow-up surveys collected 4 weeks later. Data were evaluated using descriptive analysis.

**Result:**

320 participants completed the surveys, and 288 participants were gunowners living with children. Most participants were comfortable discussing safe storage with healthcare providers and were willing to talk with friends about firearm safety. 54% reported inquiring about firearm storage in homes their children visit, 39% stored all their firearms locked-up and unloaded, 32% stored firearms/ammunition separately. 121 (37%0.8) of participants completed the follow-up survey, 84% reported using the distributed safety device and 23% had purchased additional locks for other firearms.

**Conclusion:**

Participants were receptive to firearm safe storage education by a healthcare provider and distribution of a safe storage device. Our follow up survey results showed that pairing firearm safety education with device distribution increased overall use of safe storage devices which in turn has the potential to reduce the incidence of unintentional and intentional self-inflicted firearm injuries. Providing messaging to promote utilization of safe storage will impact a firearm safety culture change.

## Introduction

Firearm injury is the leading cause of death in children and teenagers throughout the United States (1). Public health initiatives that promote tools for safe storage of firearms are essential to successfully combat this epidemic. It is estimated that 3,607 children between the ages of 0–18 years lost their lives because of a firearm in the United States in 2021 (1). Unintentional injuries in children are frequently associated with access to loaded firearms (2). The #NotAnAccident Index recorded that 2,800 unintentional injuries and deaths occurred between 2015 and 2022 due to children gaining access to firearms and these incidents occur daily (3). A national survey of parents revealed that one in three households with children had a firearm and among gun-owning households with children, approximately 2 in 10 gunowners reported storing at least one firearm in the least safe manner, loaded and unlocked (4). Furthermore gun owning families surveyed in a Southeastern United States Children’s Hospital Emergency Department, reported storing about 53% of their firearm in an unsecure manner (5). Unintentional firearm injuries primarily occur in the homes of the child victims themselves and with firearms belonging to family members (3, 6). Data show that if 20% of families who previously stored their firearms unlocked were motivated to safely store firearms securely, potentially 32% of adolescent deaths due to suicide could be prevented annually (7).

Education on the frequency of pediatric firearm injuries and the importance of safe firearm storage have been effective in increasing the likelihood that firearms will be stored securely (8, 9). Thus we sought to combat the rise in children gaining access to firearm by providing firearm safe storage education and firearm safety devices at community safety events. We hypothesized that parents would be receptive to education about firearm safety by healthcare providers and would be willing to utilize firearm safe storage devices when paired with educational intervention.

## Materials and methods

### Study setting

Data were collected by surveys at five separate community safety events in 2018 and 2019. The events included 3 safety fairs, one children’s hospital lobby tabling event and one pediatric urgent care tabling event. Two of the three safety fairs were organized by fire departments one in a suburban county the other a suburban city. The third safety fair was coordinated by an urban mental health awareness coalition. The three safety fairs organizers invited the children’s hospital injury prevention team to participate in the fair. The children’s hospital is in an urban area and the urgent care in a suburban area of the metropolitan region. Both the children’s hospital and urgent care are healthcare sites within the organization that received the grant to distribute the lockboxes. All safety fairs occurred in a region that is considered the 8^th^ largest US metropolitan area. Participants provided verbal consent before completing the in-person survey. A follow-up survey was completed via telephone at least 4 weeks later to assess the usage of storage devices given at the events. This study was approved by the Institutional Review Board of Emory University.

#### Educational intervention

Each participant who approached the giveaway event table was given an educational handout with information about the importance of safe storage along with a brief educational intervention. The educational intervention was provided by trained clinicians who were taught to explain in the detail the information that was provided in the handout. The education entailed an explaining to the participants how to safely store their firearms: unloaded, locked-up and separate from ammunition. In addition, participants with a firearm in their home and those without where all educated about the importance of asking about the presence of unsecured firearms in homes their children visited. Each participant was given a handout that was developed by the American College of Emergency Physicians (see Appendix 1).

### Inclusion and exclusion criteria

This study included a convenient sample of caregivers of anyone presenting to our booth at the community events. Caregivers voluntarily agreed to participate by approaching the table where we had signage regarding firearm safety and safe storage device giveaway. Inclusion criteria were participants who were at least 18-year-old, English-speaking with children in the home. Exclusion criteria were those without children in the home and non-English speaking. Each participant agreed to provide contact information for the follow-up survey.

### Data collection

Data were managed using REDCap, an electronic data capture tool hosted at Children’s Healthcare of Atlanta. REDCap (Research Electronic Data Capture) is a secure, web-based software platform designed to support data capture for research studies, providing (1) an intuitive interface for validated data capture; (2) audit trails for tracking data manipulation and export procedures; (3) automated export procedures for seamless data downloads to common statistical packages; and (4) procedures for data integration and interoperability with external sources (10, 11). Trained volunteers were present at the booth to assist participants with survey completion. All patient demographics were de-identified except for their phone numbers and emails. Electronic surveys were the primary source of data collection but if a paper survey was used the participants survey was then subsequently transcribed by research assistants into the REDCap software Paper surveys were used only when Wi-Fi access was inconsistent. Email and phone numbers were solely utilized to contact participants if they consented to participate in the follow-up survey. The-29-item survey included questions on demographics, presence of firearms, firearm storage and firearm safety discussions. The initial survey contained 8 demographic questions, 17 questions regarding gun ownership and secure storage and 4 questions to coordinate future surveys. The follow-up survey was brief with a total of 7 questions that focused on presence of firearms and their storage, use of storage devices given at the events, buying new storage devices, and inquiring about safe firearm storage in other homes where their children visit. Participants who consented to respond to an electronic follow-up survey received a 5$ gift card for participating. The gift card was only provided to participants once the follow-up survey was completed. The gift card was sent electronically via the provided participant email after completion of the follow-up survey. Initial survey questions and follow-up survey questions were modified from previous work done at community events by Simonetti and colleagues (8).

### Statistical analysis

Survey responses are reported as percentages. Data were analyzed using descriptive analysis. Percent difference from follow-up survey was calculated using the (follow-up value) –(initial survey value).

## Results

A total of 320 participants completed the initial survey with 288 participants identifying the number of children in their home under 18 years in their home. 32 participants did not identify the number of children in their home although they did state they had children in their home. Of the participants 39% reported firearms currently were unloaded and locked away, 32% stored their firearms and ammunition separately and 25% did not have a secure storage device for their firearms. Firearm storage or firearm presence in the homes their children visited had been inquired upon by 54% of those surveyed. Most parents were comfortable with healthcare provider education on safe storage (Table 1).

**Table 1:** Initial survey.

Initial survey, *n* = 320	Total *N*	%
Gender (Female)	184	57.5
**Age (years)**		
18–24	11	3.4
25–39	129	40.3
40–60	145	45.3
Over 60	35	10.9
**Ethnicity**		
White	124	38.8
Hispanic/Latino	21	6.6
Black/AA	155	48.4
Native American/Indian	6	1.9
Asian/Pacific Islander	1	0.3
Other	10	3.1
Children living in the home (*n* = 288)^*^		
1	82	25.6
2	94	29.4
2+	64	20.0
Have you ever inquired about firearm storage in the homes your children visit?	172	53.8
All firearms are locked-up and unloaded (yes)	126	39.4
Firearms and ammunition stored separately (yes)	102	31.9
Do you have alarm system in home (yes)	170	53.1
**What kind of safety device do you currently use?**		
Firearm safe	103	32.2
Firearm lock box	58	18.1
Trigger	57	17.8
Cable	43	13.4
Other	26	8.1
None	81	25.3
Would you be willing to talk to friends and colleagues about firearm safety?	298	93.1
Would you be comfortable discussing with healthcare providers?	292	91.3

^*^32-participants did not identify number of children in home.

Just over 1/3 (*n* = 121, 37.8%) of participants completed the follow-up survey. Of those participants, 85% had asked about the presence of firearms in the homes their children visited which is a + 23.4% rise compared to the initial survey. When asked if all their firearms were stored locked and unloaded there was a + 27.6% increase in storing firearm securely. Furthermore, 23% of participants had purchased additional locks for other firearms (Tables 2, 3).

**Table 2:** Follow-up survey.

Follow up survey, *n* = 121	Total *N*	
Have you inquired about the presence of unlocked firearms in the homes that your children frequent?	85	70.2
Did you find it to be an easy conversation to initiate?	101	83.5
Are all your firearms locked and unloaded?	81	66.9
Is all your ammunition stored locked?	86	71.1
Is your ammunition stored separately from your firearm?	78	64.5
**Did you buy any firearm locks for your other firearms?**		
Yes	28	23.1
No	41	33.9
N/A	37	30.6
Have you been using the device that was distributed at the Safety Fair? Yes	102	84.3

**Table 3:** Comparison of participants.

Additional questions	Initial survey (*n* = 320)	Follow up survey (*n* = 121)	Percentage change
Do you inquire about firearms in homes that your children visit?	172 (53.8)	85 (77.2)	23.4
Are all your firearms stored locked and unloaded?	126 (39.4)	81 (66.9)	27.5
Are your firearms and ammunition stored separately?	195 (60.9)	78 (64.5)	3.6

## Discussion

### Healthcare providers recommendation a valuable tool for safe storage promotion

This study demonstrates that parents presenting to a community safety event, overwhelmingly appear comfortable with healthcare providers offering guidance on safe storage of their firearms (91%). This is important to note as many healthcare providers feel parents would not be receptive and therefore are reluctant to educate. But studies have consistently shown that both gun owners and non-gunowners feel that it is appropriate for physicians and other healthcare professionals to provide gun safety education (12). Some physicians also report feeling that it is forbidden to have these conversations by either legislation or HIPAA. Laws that have previously proposed such as a law in Florida that was enacted in 2011 have been struck down (13). Furthermore, there are not any provisions in Health Insurance Portability and Accountability Act or Affordable Care Act (ACA) that states that healthcare professionals cannot talk about gun safety. In fact, within the ACA there are requirements for the collection of firearm information by “wellness and health promotion” programs (14). Furthermore, after an increase in firearm-related injuries presenting to EDs in 2010–2019, and an unprecedented increase in firearm injuries occurring in 2020 (15, 16) the American Academy of Pediatrics released an expansive policy statement encouraging pediatricians to educate parents about firearm safety (17). Thus, our community firearm injury prevention program is actually encouraged by federal law and the largest professional organization of physicians who care for children.

### Safety education as a tool for behavioral modification

During the initial survey, 54% of participants reported that they inquired about firearm storage in the homes their children frequented. This increased by 16.5% when reassessed in the 4-week follow-up survey. This highlights the potential impact of educational intervention geared toward parents that can motivate a culture change and reduce access to unsecured firearms and therefore unintentional injuries. Our study revealed that parents are potentially amenable to change in behavior with 84% of our participants finding it easy to have conversations about unlocked firearms in homes that their children visit after the four-week follow up. While there was a general script as described in methods (educational intervention) that was followed for counseling at each of the five events there were numerous volunteers whose diversity of age, gender and experience impacted the way the survey was administered but our results suggest that interacting at the safety fairs may have influenced parents to inquire about other opportunities where their children may have access to loaded firearms. Previous work by our group and others has shown that after receiving education on the importance of asking about unsecured firearms most participants report feeling comfortable asking if there were a firearm in homes prior to their child’s visit (18).

Decreasing youth access to firearms is important as our local data shows that at least 14% of teens could access a firearm within 24 h (5). A national survey of parent–child dyads described even greater access and demonstrated that while 70% of parents believed their child would not be able to access a firearm, 37% of adolescents said they could within an hour (19). One study that demonstrates the ineffectiveness of just telling children not to handle a firearm was performed at our institution where boys were left in a room with a firearm that was engineered for safety unbeknownst to the participants; 76% of the boys handled the firearm and 48% pulled the trigger despite most of the participants previously receiving firearm safety instructions (20). These studies highlight the importance of parental awareness of the significance of safe, secure consistent locked up storage of firearms. One probability study depicted that up to 32% of unintentional and suicide related firearm deaths could be reduced with motivating caregivers to store firearms safely (7).

### Encouraging results of parental utilization of safety device

After the four-week follow-up, 102 of 121 (84%) of participants reported using the safe storage device they were given. In addition, there was an increase of participants who reported having all their firearms locked and unloaded: 39% (126, *n* = 240) during the initial survey and 76% (81, *n* = 121) during follow-up 4 weeks later. This is consistent with other studies that have shown effectiveness of interventions to promote safe storage that are paired with counseling and distribution of safe storage device compared to interventions without distribution of safe storage device (9). Our group distributed free safe storage lock boxes or trigger locks which have been established to be the preferred methodology over providing devices at a reduced cost (21). In addition other studies have indicated that parents would be more inclined to use a firearm lock box than cable lock or trigger lock (22). This may be because families are increasingly purchasing firearms for their protection, as 72% of US gun owners cite security as their major reason to own a firearm and lock boxes add ready access to their firearms (23). To our knowledge this study is one of the only studies to gauge receptiveness to health care provider delivered messages on firearm safety at a community-based safety event. Furthermore, to our knowledge, we are the only program describing this type of education in the Southeastern United States. It is a crucial area to target it in a region with historically high gun ownership and therefore above average rates of unintentional pediatric firearm injury (8, 24–26).

### Limitations

This study has some limitations. First, only participants who spoke English were approached to complete the surveys. This may have introduced a sampling bias and thus is not representative of all families who own firearms in our region or in the United States. Second, this study used a convenience sample as only those who visited our booth at the safety fair events were included. Caregivers who attend a fair that is focused on health and well-being may be preconditioned to adhere to and or be more receptive to the education that was provided. Thus, self-selection probably occurred and is a major limitation to this study, but the lockboxes were free thus open to all who approached our table and met inclusion criteria. Third, self-reported surveys have potential for social desirability bias this was mitigated by writing clear concise non-leading questions many questions giving respondents the opportunity to free text responses on some questions. Fourth, there was a reduced number of initial survey participants who consented to follow-up survey contact and subsequently proceeded to complete the follow-up survey which could lead to nonresponse bias. We opted to incorporate a more diverse array of survey participants and a broader perspective by integrating findings from both the initial and follow-up surveys. This decision was made despite not obtaining consent from all initial survey respondents for the follow-up survey. This approach left the study open to a significant non-response bias, the extent of which remains uncertain.

Fifth, with follow-up after only 4 weeks, we were not able to assess the long-term effectiveness and sustainability of our intervention measures.

Finally, this study relied on self-reported follow-up responses and may not reflect actual practices, however, the receptivity of onsite education and receiving a safety lock was demonstrated. Future large-scale studies conducting long-term follow-up research to track the impact of utilization of firearm safe storage and education on reducing unintentional firearm injury are supported by this pilot study.

## Conclusion

Our findings support previous studies that show participants with children in their household are receptive to education on firearm storage and using firearm safety devices. Furthermore, it underlines the efficacy of pairing counseling with safe storage device distribution. Unintentional and self-inflicted intentional injury in children due to unlocked or loaded firearms stored unsafely can be mitigated through education, counseling, and safety devices. Therefore, more research is needed to determine effective methods of dissemination. Continued research can focus on evaluating different dissemination strategies, assessing their impact on various demographics, and identifying barriers to implementation. Additionally, longitudinal studies tracking the outcomes of households that receive education, counseling, and safe storage devices can provide valuable insights into the long-term effectiveness and sustainability of these interventions. Conducting more research in this area can assist policymakers, healthcare professional, and educators that inform evidence-based programs and policies aimed at reducing firearm-related injuries and death in youth.

## Data availability statement

The raw data supporting the conclusions of this article will be made available by the authors, without undue reservation.

## Ethics statement

The studies involving humans were approved by Emory University Institutional Review Board. The studies were conducted in accordance with the local legislation and institutional requirements. The participants provided their written informed consent to participate in this study.

## Author contributions

KF: Conceptualization, Data curation, Formal analysis, Funding acquisition, Writing – original draft, Writing – review & editing. ZB: Writing – original draft, Writing – review & editing. TG: Data curation, Formal analysis, Writing – review & editing. JJ: Conceptualization, Investigation, Methodology, Project administration, Writing – review & editing. Data curation. ABo: Conceptualization, Investigation, Methodology, Project administration, Writing – review & editing, Data curation. RW: Conceptualization, Methodology, Writing – review & editing. ABh: Conceptualization, Data curation, Funding acquisition, Investigation, Writing – review & editing. MS: Methodology, Writing – review & editing, Project administration. HS: Conceptualization, Supervision, Visualization, Writing – review & editing.
