# Supplementary material for: Receptivity of providing firearm safety storage devices to parents along with firearms safety education
Source: Front Public Health. 2024 Mar 21;12:1352400. doi: 10.3389/fpubh.2024.1352400 (PMC10991684; doi:10.3389/fpubh.2024.1352400)
Supplement: Supplementary file 1 [file Data_Sheet_1.PDF]

ASK one question, it could save your child's life.

ASK: "Is there an unlocked gun where my child plays?"

1 in 3

homes with children have guns, many left unlocked or loaded

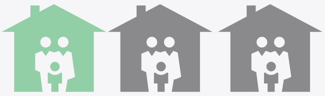

nine

children and teens are shot each day in gun accidents

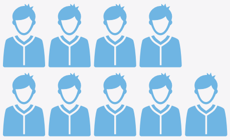

80%

of unintentional firearm deaths of kids under 15 occur in a home

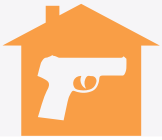

If the answer is no, then you have one less thing to worry about.

If you ever have a concern about gun safety in a house where your child plays, invite the kids to play at your house instead.

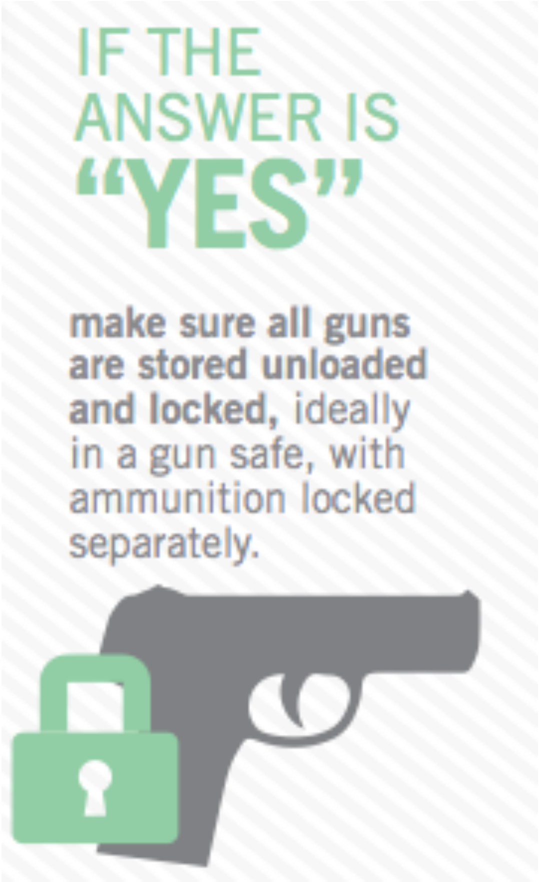

Hard conversations come with being a parent. But one could save your child's life.

TIP: Integrate the question into a conversation that includes other questions you might ask: "Will they watch television? Will they be eating certain foods? Will they be on the internet? IS THERE AN UNLOCKED GUN IN THE HOME?"

For more information: www.askingsaveskids.org or www.acep.org
